# Rib Exostoses Presenting as Mediastinal Masses: A Rare Presentation and Minireview of the Literature

**DOI:** 10.1155/2020/8283565

**Published:** 2020-01-09

**Authors:** Doina Butcovan, Veronica Mocanu, Raluca Ecaterina Haliga, Dana Baran, Carmen Ungureanu, Ştefana Carp, Grigore Tinică

**Affiliations:** ^1^Department of Morpho-Functional Sciences, “Gr. T. Popa” University of Medicine and Pharmacy, Iasi, Romania; ^2^Department of Pathology, “Prof. George Georgescu” Institute of Cardiovascular Diseases, Iasi, Romania; ^3^Department of Medical Sciences III, “Gr. T. Popa” University of Medicine and Pharmacy, Iasi, Romania; ^4^Department of Cardiovascular Surgery, Institute of Cardiovascular Diseases “Prof. George Georgescu”, Iasi, Romania

## Abstract

Costal osteocartilaginous exostoses, also known as osteochondromas, are the most common neoplasms of the long bones but are rare tumors of the ribs. Osteochondroma is often asymptomatic and incidentally observed. Tumors typically begin to grow before puberty and continue until bone maturation is reached. Our paper presents the case of a 16-year-old young male who was admitted to the hospital with nonspecific symptoms and having a family history of exostosis. Chest X-ray and computed tomography imaging revealed multiple costosternal exostoses, manifested as mediastinal masses, with protrusion into the thoracic cavity, exerting compressive effects on the ascending aorta and pulmonary parenchyma. Surgery is required in childhood if lesions are painful. But if tumor formation occurs in adulthood, such pathological bony outgrowths should always be resected for avoiding further complications. In this patient, surgical intervention removed the tumoral masses and improved the symptoms. Subsequently, histological exam confirmed the diagnosis of osteocartilaginous exostoses and showed the lack of dysplastic changes.

## 1. Introduction

Osteochondroma (OC) is a benign tumor of the bone surface. It appears as a subperiosteal osseous excrescence, which projects above the bone surface (exostosis), being composed of mature bone and a cartilaginous cap. As a rule, osteochondromas are located in the metaphysis of long bones, where a portion of the epiphyseal growth cartilage herniates through the periosteal bone surface [1].

OC is a common osteocartilaginous tumor of the long bones, but rare tumors of the ribs, predominating in males (male : female ratio, 2 : 1). These tumors develop during childhood, a period of the fastest bone skeletal growth, and once formed, they remain for the rest of the individual's life. Most cases are diagnosed in the first two decades of life and are frequently found incidentally [2]. Usually, OC tumors are solitary, rarely occurring as multiple hereditary exostoses (MHE), both being associated with mutations in EXT1 (8q24) and EXT2 (11p11) genes [3, 4].

Histologically, OC is composed of mature bone, including bone marrow spaces, with a cartilaginous cap. The cortex and spongiosa of the tumoral mass communicate with those of the host bone [2]. Correlations between imagistic aspects and histopathological exam are often specific. Radiologically, the tumoral growth direction is disposed perpendicular to the long axis of the bone. A mature bony tumor is continuous with the cortex; the tumoral trabecular bone communicates with the trabecular network of the underlying bone. The tumor has a thin, lobulated cartilaginous cap, which may contain calcifications. If the lesion is not painful, the tumor is treated conservative. Surgical resection is recommended for definitive diagnosis and also for prevention of complications and malignancy [5].

We present the case of a young patient with multiple costal exostoses displaying as mediastinal masses within the left hemithorax, which exerted pressure on the first part of the aorta and pulmonary parenchyma.

## 2. Case Report

A 16-year-old patient was admitted at “Prof. George Georgescu” Cardiovascular Disease Institute, Iasi, Romania, in January 2018, for dyspnea to moderate exertion and fatigue. One month ago, in a pediatric clinic, he had been diagnosed with multiple exostoses disease manifested as mediastinal masses. At the same time, a well-known family history of exostosis was also documented.

Clinically, patient chest's deformation of carinatum type was noted. Lab tests indicated normal hemoleucogram, hepatorenal function, and blood glucose level and absence of inflammatory syndrome. Echocardiography revealed normal heart cavities and function.

**Figure 1:**
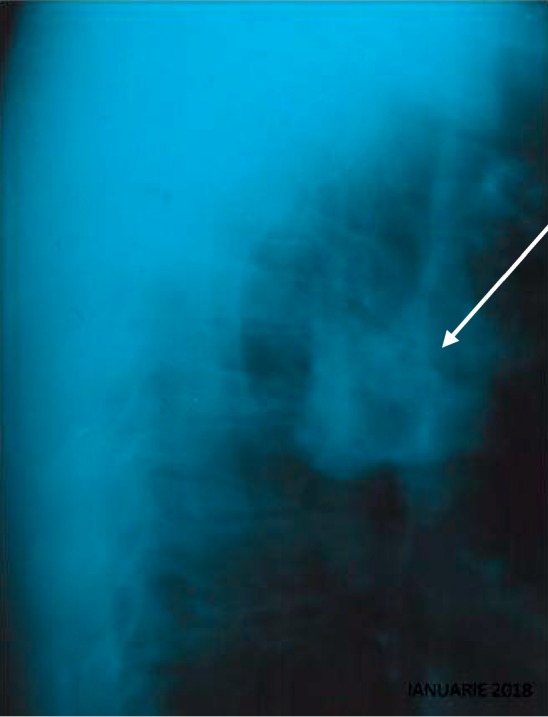
Profile lateral chest X-ray showing a retrosternal mass (arrow).

The chest radiograph showed bony lesions and retrosternal mass in the lateral view (Figure 1), opacities of chalky intensity beingidentified on the left side of the thoracic wall at the level of 2^nd^, 3^rd^, and 4^th^ left costosternal joints.

**Figure 2:**
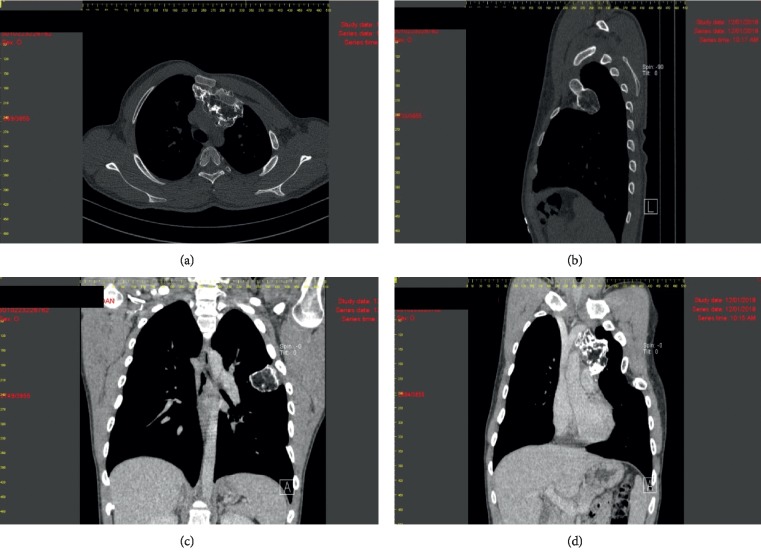
Thoracic computed tomography (CT) scan. (a) Osteochondroma developed on the internal face of left ribs, anterior arch, with mass effect on anterior mediastinum, which is associated with rib cage deformation (axial section, bone window). (b) Osteochondroma developed on the internal face of left ribs, anterior arch (sagittal section, bone window). (c) Osteochondroma developed on the internal face of left ribs, lateral arch (coronal section, bone window). (d) Osteochondroma developed on the anterior arch of left ribs, with preaortic expansion in the retrosternal space and ascending aorta compression.

Thoracic computed tomography (CT) scan (Figure 2) revealed a expansive heterogeneous mediastinal mass with appearance of the cortical and medullar bone, at the level of the left 2^nd^ and 3^rd^ costosternal joints, with preaortic expansion in the upper retrosternal space and ascending aorta compression. Also, CT described exostoses developed toward the pulmonary parenchyma, located at the level of the 3^rd^ and 4^th^ ribs. Small focal pulmonary areas with a “matt glass” appearance, perhaps due to hypoventilation, were visible in the vicinity of the bone masses described above.

**Figure 3:**
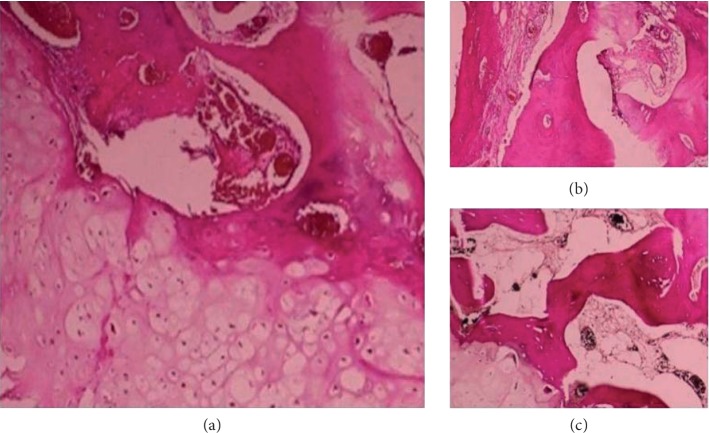
(a) Normal bone covered by normal cartilage; (b) mature cortical bone covered by fibrous tissue; (c) spongy bone and bone marrow spaces.

Histopathological exam (Figure 3) of the samples collected during surgical intervention revealed osteocartilaginous exostoses, with mature cortical bone covered by fibrous tissue and areas of spongy bone and bone marrow spaces, aspects characteristic for osteochondroma. No malignant changes were described.

Surgery consisted of removing tumoral masses and costosternal joints. The postsurgical evolution of the patient was favorable, with improving clinical conditions, ameliorating symptoms of dyspnea and fatigue.

## 3. Discussion

Costal OC is a very rare osteocartilaginous exostosis of the rib, composed of cortical and medullary bone, with an overlying hyaline cartilage rim. Majority of osteochondromas occur as solitary lesions in young male patients. Multiple exostosis affects at earlier ages in the first decade of life, occurring in the case of MHE [2, 6]. Our patient also had a familial history of OCs.

About 3% of solitary OCs are of vertebral and costal origin, while 7% of individuals have multiple exostoses [3, 4]. Costal OC may present as a compressive mass in the chest wall. The main reasons for presenting our patient to Cardiovascular Disease Institute were multiple exostoses disease manifested as mediastinal masses, identified in another hospital, clinically expressed by dyspnea and fatigue.

Usually, the diagnosis is based on clinical and radiological documentation, supplemented, if available, by histological evaluation of OCs [2]. Clinically, OC is often asymptomatic and represents an incidental radiographic finding. Symptoms arise as a result of location, size, and pressure effects on adjacent structures. Symptomatic patterns are either due to mechanical effects (chest compressive mass on nerves or vessels) of the tumoral mass [5] or malignant transformation [2].

Chest radiography may be the only imaging study required, but, in the mediastinal location, only the use of CT can demonstrate cortical and medullary continuity between osteochondroma and parent bone [1, 7]. Histopathologically, the tumor has a rim composed of mature hyaline cartilage enclosed in a fibrous tissue and centrally contains islands of mature bone; bone marrow elements are present within the bony mass [8]. These aspects were found in the biopsy sample of our patient, confirming the diagnosis of osteochondroma.

Multiple mediastinal OC should be distinguished from heterotopic ossification, Ollier and Maffucci diseases, and osteofibrous dysplasia (OFD).

Heterotopic ossification (HO), also known as heterotopic bone formation, represents the presence of bone in soft tissue, where bone normally does not exist. Acquired HO is generally related to trauma and spinal cord or central nervous system injury, resulting from metaplastic changes and osteoid formation in the mediastinum [9]. This condition should not be confused with metastatic calcification, produced by hypercalcemia, or dystrophic calcification, which occurs in debris tissues. Bone scanning and other imaging tests are frequently used to distinguish between these diagnostic possibilities.

Ollier disease is a rare nonhereditary sporadic disorder, where intraosseous benign cartilaginous tumors (enchondromas) develop close to the growth plate cartilage. Ollier disease carries a high risk of skeletal malignancy [10]. Another related illness, Maffucci disorder, is characterized by enchondromas associated with multiple hemangiomas, which usually occur in hands and feet. Maffucci syndrome also carries a higher risk for cancer [11].

Osteofibrous dysplasia (OFD) is histologically similar to fibrous dysplasia, except that fibroblast proliferation surrounds islands of woven bone with osteoblastic rimming. Mitotic figures are absent, as well [12].

A study of the available literature data made on 1,051 cases of MHE found that common complications include impaired articular function, angular deformities, impingement on neighbouring tissues, and also chondrosarcoma transformation in rare cases [13]. Other possible evolution of OC involves unusual complications of costal exostoses, such as spontaneous hemothorax, pneumothorax, and pericardial effusion [14–16]. There are also rare cases of acute coronary syndrome, secondary to intermittent extrinsic compression of the left anterior descending coronary artery by inward-pointing rib exostosis [17]. Fortunately, the patient did not present any complications mentioned above, even if the exostoses were multiple.

Regarding the prognosis, OC is a benign tumor. Solitary lesions have maximum 1-2% risk of malignant transformation [2], while MHE are at higher risk (overall values 5–25%) [2, 6]. Malignant transformation may occur as low-grade secondary chondrosarcoma. MRI can be useful in differentiating benign from low-grade malignant cartilaginous tumor [7].

Related to tumor management, OC growth often stops at skeletal maturity and may spontaneously regress. In most instances, no treatment is required. Surgery remains the treatment of choice when OCs are symptomatic. The purpose of surgical intervention is to prevent complications and to reduce the risk of malignancy [5, 18]. So, once removed, OCs should be examined for malignancy.

## 4. Conclusion

This is an interesting case of a young male presenting with nonspecific symptoms, having a family history of exostosis. Chest X-ray and computed tomography imaging revealed multiple costosternal exostoses, manifested as mediastinal masses, with protrusion into the thoracic cavity, exerting compressive effects on the ascending aorta and pulmonary parenchyma. A particular aspect in this case is that usually the ribs and costosternal joints are rare locations of OC. Surgical intervention removed the tumoral masses and improved the symptoms. Histopathological examination of excised masses excluded malignization of exostoses.
